# Wastewater surveillance as an early warning indicator for norovirus: Validating wastewater signals against clinical indicators in British Columbia, Canada, 2021–2025

**DOI:** 10.14745/ccdr.v52i78a04

**Published:** 2026-07-30

**Authors:** Bridget Irwin, Natalie Prystajecky, Mayank Singal, Sharon Relova, Anya Smith, David McVea

**Affiliations:** 1 Canadian Field Epidemiology Program, Centre for Emergency Preparedness, Public Health Agency of Canada, Ottawa, ON; 2 British Columbia Centre for Disease Control, Vancouver, BC; 3 Department of Pathology and Laboratory Medicine, University of British Columbia, Vancouver, BC; 4 School of Population and Public Health, University of British Columbia, Vancouver, BC

**Keywords:** norovirus, gastrointestinal illness, wastewater surveillance, wastewater-based epidemiological monitoring, public health surveillance, validation study, early-warning indicator

## Abstract

**Background:**

Norovirus is the leading cause of acute gastroenteritis globally and causes significant morbidity. Despite this, norovirus is not reportable to the public health system in most jurisdictions, making it challenging to detect and control local transmission. Wastewater-based epidemiology is a potential tool for narrowing this gap, allowing for non-invasive, population-level surveillance.

**Objective:**

In this study, it assess wastewater-based epidemiology as a surveillance tool for monitoring norovirus in British Columbia, Canada, by comparing norovirus wastewater signals to norovirus-related hospital admissions and genogroup I (GI)-related emergency department (ED) visits.

**Methods:**

More than six thousand (n=6,167) wastewater samples from 12 municipal treatment plants were collected between January 10, 2021–September 27, 2025 and pooled to generate a population-weighted average for the province. Norovirus hospital admissions and GI-related ED visits were extracted for the same period from the Discharge Abstract Database and the National Ambulatory Care Reporting System, respectively, and were compared with wastewater signals using time-lagged cross-correlation analyses.

**Results:**

Time-lagged cross-correlation analyses indicate significant positive correlation overall between unadjusted norovirus wastewater concentrations and norovirus hospitalizations (Spearman’s ρ=0.69, *p*<0.01) and GI-related ED visits (Spearman’s ρ=0.84, *p*<0.01), with wastewater leading hospitalizations by two weeks (Spearman’s ρ=0.71) and aligned with ED visits (Spearman’s ρ=0.83).

**Conclusion:**

The findings support the validity of using wastewater-based epidemiology for monitoring norovirus at the population-level and suggest that it may provide an early warning signal for severe norovirus activity requiring hospitalization, prompting a more timely response.

## Introduction

Norovirus is the leading cause of acute gastroenteritis worldwide and causes significant illness (1,2), estimated at over 677 million cases globally per year (1,2), including over three million in Canada (3). Spread through fecal-oral or vomit-oral routes, norovirus presents with rapid onset of nausea, vomiting and diarrhea and is highly infectious, with fewer than 100 viral particles required to cause illness (4). This low infectious dose, combined with extended and asymptomatic viral shedding, short-lived immunity and strong environmental persistence, results in efficient transmission and frequent outbreaks (4).

While most norovirus cases resolve within one to two days with supportive treatment, young children, the elderly and people with medical co-morbidities may develop complications resulting in hospitalization or death (4,5). Globally, norovirus is estimated to cause over 200,000 deaths and to cost $60.3 billion USD each year (over $80 billion CAD) due to healthcare costs and productivity losses (2,6). In Canada, hospitalization costs are estimated to exceed $21 million CAD (7).

Despite its health and economic impacts, norovirus is not considered a disease of public health significance in most jurisdictions, including in British Columbia (BC), Canada, where reporting is only required for facility-based outbreaks and laboratory confirmation is not mandatory. Although the national incidence of laboratory confirmed norovirus cases is collected and reported weekly by the National Enteric Surveillance Program, a 2014 to 2015 study found that just 14.5% of Canadians experiencing acute gastroenteritis sought medical care, and only 12.5% of those were ordered to undergo diagnostic testing (8). This low rate of health care seeking and testing means that the disease is systematically underrepresented in surveillance databases and its true burden is difficult to assess.

Wastewater-based epidemiology (WBE) gained prominence during the COVID-19 pandemic as a valuable public health surveillance tool, particularly in situations where clinical testing is limited. Wastewater-based epidemiology involves extracting viral information from fecal matter and offers several benefits over traditional surveillance, including that it provides a non-invasive, population level estimate of disease prevalence (9–12) and that it captures both symptomatic and asymptomatic infections (11,13). Wastewater-based epidemiology may also have economic benefits and has been shown to be more cost-effective than clinical surveillance in some settings, particularly those with high disease severity, minimal routine clinical surveillance and high intervention effectiveness (11,14,15). Furthermore, there is evidence to suggest that WBE can detect some viruses prior to symptom onset. This may allow it to be used to predict disease activity in the community and guide interventions, though the extent of this lead time varies across temporal and geographic contexts (10,11,13,16,17).

Given its success for COVID-19, WBE is increasingly being adapted to monitor other pathogens such as norovirus. Indeed, WBE has the potential to improve norovirus surveillance by allowing for routine monitoring of the burden of illness and temporal trends in the community, as well as providing valuable information on the emergence of new outbreaks and variants of the disease. Such information can guide public health actions to reduce transmission and may also provide an early warning to healthcare facilities, supporting preparedness efforts and the implementation of preventative measures.

Wastewater-based epidemiology for norovirus has been explored globally (18–29) and several studies have found that wastewater signals correlate relatively well with both outbreak counts (18,29–31) and individual case counts (21,23,31,32); however, few researchers have investigated long term trends in these relationships and little work has been done to explore WBE as an early warning indicator. Given these gaps and building on previous work, this study seeks to evaluate WBE as a tool for monitoring severe norovirus in BC by assessing correlation between norovirus concentrations in wastewater and clinical indicators over a multi-year period.

## Methods

### Wastewater sample collection and processing

Between January 10, 2021 and September 27, 2025, influent wastewater samples were collected one to five times per week from 12 predominantly urban wastewater treatment plants (WWTPs) across BC. Combined, these facilities treat the waste of approximately 57% of the population (3.2 million people), including the majority of Metro Vancouver. At each treatment plant, 24-hour composite samples were collected using autosamplers and one litre of coarsely screened raw influent was extracted. Samples were refrigerated prior to shipping and were transported in coolers on ice to the BC Centre for Disease Control laboratory within 48 hours of collection for immediate processing. Wastewater processing methods are described in full in **Appendix**, **Supplemental material**. Briefly, samples were homogenized and concentrated by centrifugal filtration before being subjected to automated RNA extraction. Extracted ribonucleic acid (RNA) was tested for the presence of genogroup I (GI) and genogroup II (GII) norovirus using multi-plex real-time quantitative polymerase chain reaction (RT-qPCR), with a spiked in synthetic DNA used as an inhibition control. A standard curve generated with synthetic DNA was used to quantify norovirus in the samples. Assays demonstrated high linearity with a lower limit of detection of cycle threshold (CT) 35, and viral concentrations were calculated as genome copies per millilitre of wastewater using RT-qPCR results and sample-specific conversion factors.

### Clinical data

Hospitalization data for BC residents were extracted from the Discharge Abstract Database (DAD) for the time period, January 10, 2021 to September 27, 2025. All 81 acute care facilities in BC must report to the DAD, which captures up to 25 diagnoses per admission using International Classification of Diseases, 10^th^ revision, Canada (ICD-10-CA) codes (33). Cases were included in this study if a diagnosis of acute gastroenteropathy due to norovirus (ICD-10-CA code A08.1) was recorded in any position. Emergency department (ED) visit data for BC residents were extracted for the 30 BC EDs included in the National Ambulatory Care Reporting System (NACRS) during the same time period. The NACRS includes data on approximately 76.4% of all provincial ED visits and captures up to three complaints per visit using Canadian Emergency Department Information System (CEDIS) codes (34). Cases were included in this study if a presenting complaint of diarrhea (CEDIS code 254) or nausea and/or vomiting (CEDIS code 257) was recorded in any position.

### Statistical analyses

Statistical analyses and data visualizations were performed using R Studio version 2023.06.1 (35). For ease of comparison, clinical and wastewater data were down sampled to a single measurement for each epidemiological week of the study to create an evenly spaced dataset with 246 observations. For hospitalizations and ED visits, cases were summed per epidemiological week of presentation. For the wastewater samples, viral concentrations of GI and GII were summed to determine the overall norovirus load and the resulting values were normalized by the WWTP’s daily flow rate to adjust for variation in day-to-day wastewater volume using the following formula:

virus copies per litre × flow in litres per day =virus copies per day

Weekly weighted average norovirus concentrations were then calculated to account for WWTPs serving different percentages of the population and sampling occurring at different frequencies, as described in Appendix, Supplemental material.

The data were not normally distributed, so non-parametric measures of analysis were used. First, crude correlation between norovirus wastewater concentrations and clinical indicators was assessed using Spearman’s rho coefficient (ρ) (36). Next, time lagged cross-correlations were calculated to determine at what shift peak synchrony occurred between the wastewater concentrations and the clinical indicators. Positive time lagged cross-correlation lag times indicated that wastewater signals preceded the clinical indicators, while negative lag times indicated that wastewater signals lagged the clinical indicators (37,38). Time lagged cross-correlations were generated using the ccf_boot function in the R package “funtimes” (37), which accounts for potential autocorrelation in wastewater concentrations and clinical indicators by repeatedly resampling the data to ensure the results are reliable (37,39). All analyses were performed both on the unadjusted wastewater data and on wastewater data normalized for flow. Locally estimated scatterplot smoothing with a span of 0.02 was applied to both the wastewater and clinical timeseries data to reduce short-term noise while preserving overall trends.

## Results

A total of 6,167 influent wastewater samples were collected over the study period, of which 94.3% were positive for norovirus GI and 99.9% were positive for norovirus GII. During the same period, there were 1,252 hospital admissions related to norovirus (ranging from 0–24 admissions per week), where norovirus was the most responsible diagnosis for 46.3% (median position=2; interquartile range [IQR]: 3) and 237,365 ED visits related to GI symptoms (ranging from 595–1,502 visits per week), where diarrhea or nausea/vomiting was the primary presenting complaint for 93.4% (median position=1; IQR: 0). The ages of the individuals admitted to hospital and presenting to the ED are summarized in **Table 1**. Hospitalizations were a mean of 63.04 years (± 27.36), while ED visits were a mean of 35.98 years (± 27.38).

**Table 1:** Age characteristics of norovirus-related hospital admissions and gastrointestinal-related emergency department visits in British Columbia, 2021–2025

Age group (years)	Hospital admissions n (%)	Emergency department visits n (%)
0–4	61 (4.87%)	42,329 (17.83%)
5–11	49 (3.91%)	20,390 (8.59%)
12–18	34 (2.72%)	11,800 (4.97%)
19–29	45 (3.59%)	36,014 (15.17%)
30–39	70 (5.59%)	29,320 (8.59%)
40–49	51 (4.07%)	19,827 (8.35%)
50–59	88 (7.03%)	19,545 (8.23%)
60–69	177 (14.14%)	21,005 (8.85%)
70–79	233 (18.61%)	19,276 (8.12%)
80 and older	421 (33.63%)	17,840 (7.52%)
Unknown	23 (1.84%)	19 (0.01%)
Total	1,252	237,365

**Figure 1:**
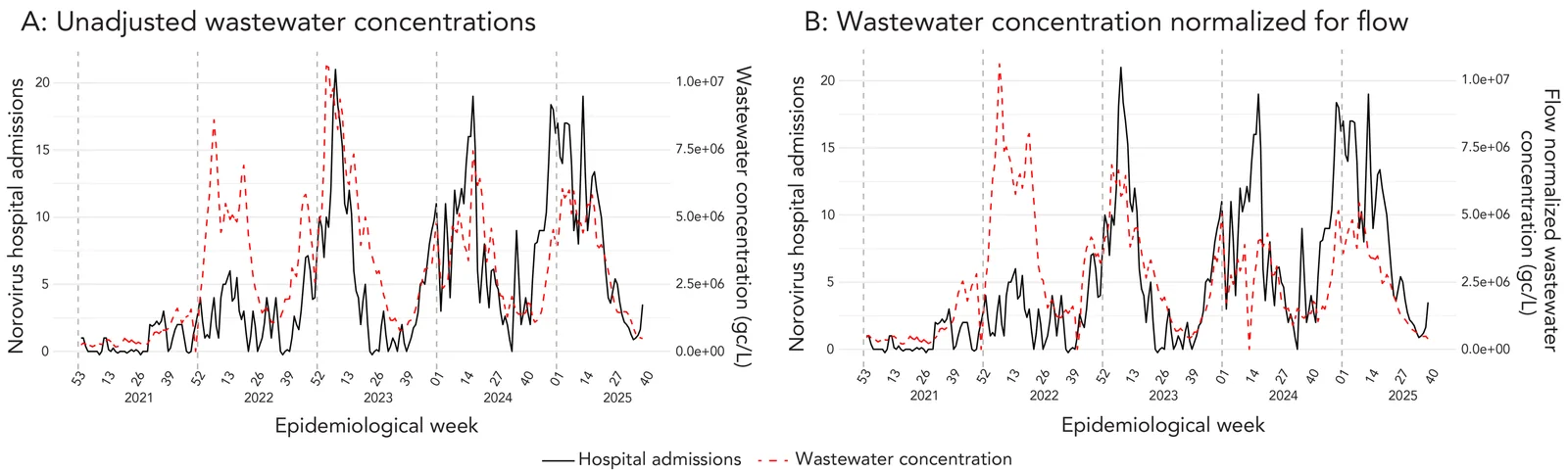
Weekly norovirus hospital admissions and norovirus wastewater concentrations in British Columbia, 2021–2025

**Figure 2:**
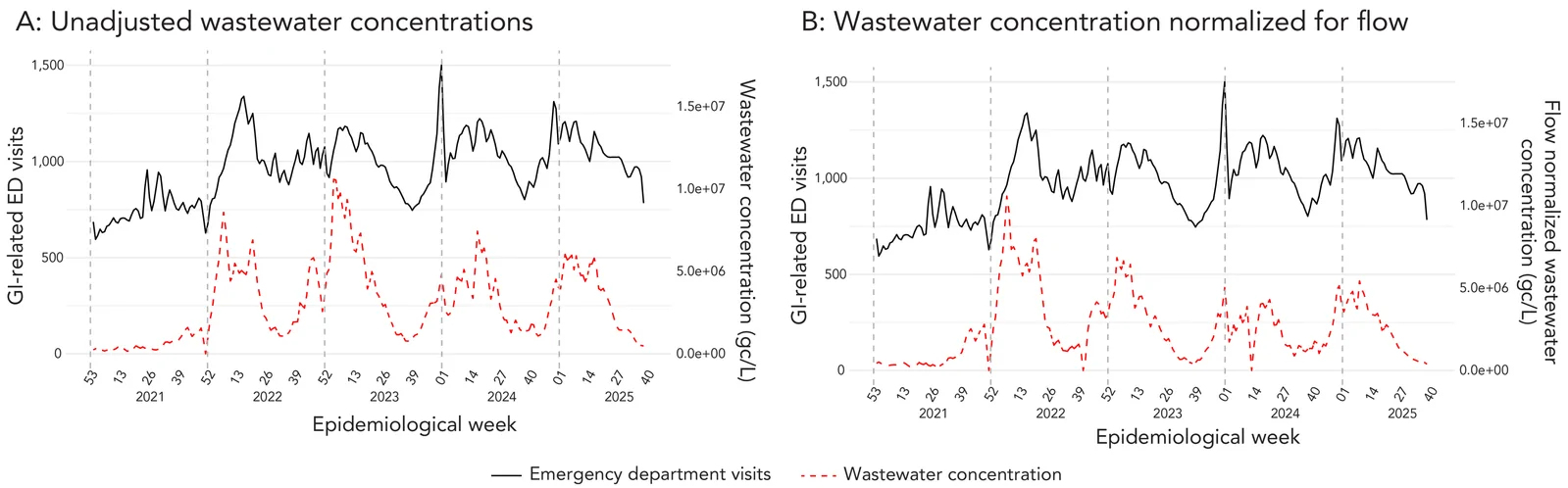
Weekly gastrointestinal-related emergency department visits and norovirus wastewater concentrations in British Columbia, 2021–2025 Abbreviations: ED, emergency department; GI, genogroup I

Overall, trends in norovirus wastewater mirrored hospitalizations and ED visits, with wastewater and clinical indicators highest in the winter and spring each year and lowest in the summer and fall for both unadjusted (**Figure 1**, **Figure 2**) and flow-normalized (Figure 1, Figure 2) wastewater. Crude correlation analyses showed moderate-strong positive correlation between norovirus concentrations and hospitalizations (Spearman’s ρ=0.69; *p*<0.001) and ED visits (Spearman’s ρ=0.84; *p*<0.001), and these findings held when wastewater was normalized for flow (hospitalizations: Spearman’s ρ=0.62; *p*<0.001; ED visits: Spearman’s ρ=0.76; *p*<0.001).

**Figure 3:**
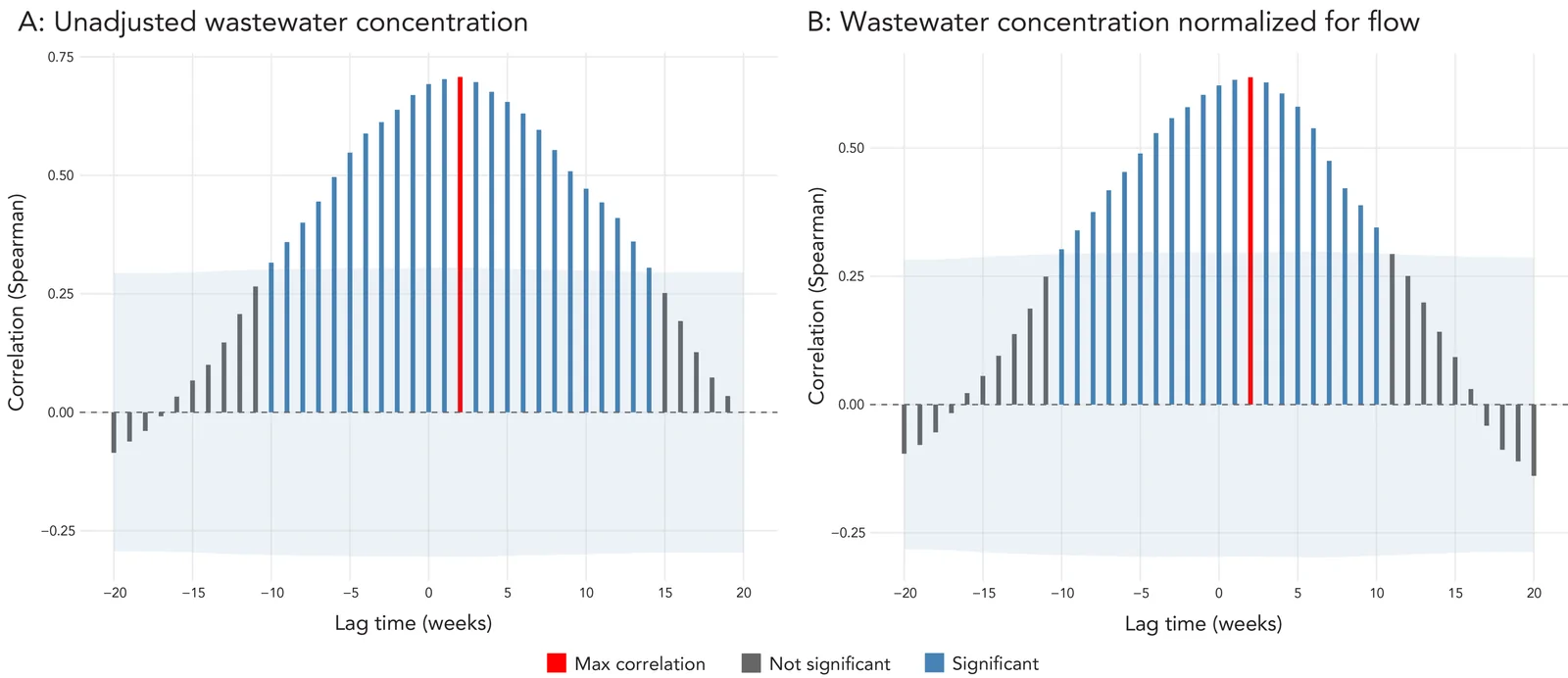
Bootstrapped time-lagged cross correlation between norovirus hospital admissions and norovirus wastewater concentrations in British Columbia, 2021–2025 Note: The lag that produces the highest Spearman’s R (correlation coefficient) is shown in red, while significant correlations shown in blue. Bootstrapped cross-correlation distributions generated under the null hypothesis of no association are represented by the shaded grey area

**Figure 4:**
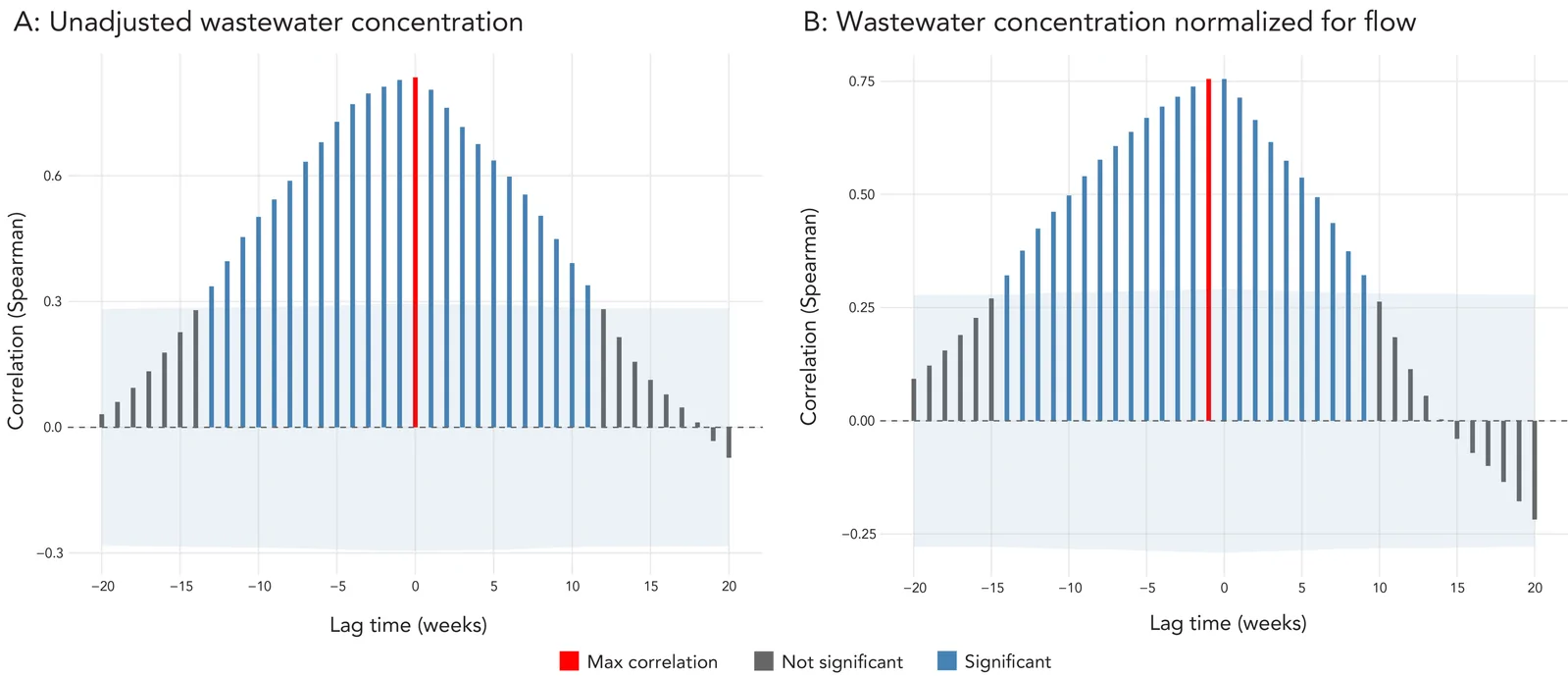
Bootstrapped time-lagged cross correlation between gastrointestinal-related emergency department visits and norovirus wastewater concentrations in British Columbia, 2021–2025 Note: The lag that produces the highest Spearman’s R (correlation coefficient) is shown in red, while significant correlations shown in blue. Bootstrapped cross-correlation distributions generated under the null hypothesis of no association are represented by the shaded grey area

Time lagged cross-correlation analyses were performed to assess the temporal relationships between wastewater concentrations and hospitalizations (**Figure 3**) and ED visits (**Figure 4**). The strongest correlation between the wastewater signal and hospital admissions occurred when wastewater preceded admissions by two weeks (unadjusted wastewater: peak synchrony=2, Spearman’s ρ=0.71; flow-normalized wastewater: peak synchrony=2, Spearman’s ρ=0.64). The strongest correlation between the wastewater signal and ED visits occurred when the signals were not shifted (unadjusted wastewater: peak synchrony=0, Spearman’s ρ=0.83), or when wastewater lagged ED visits by one week (adjusted wastewater: peak synchrony=−1, Spearman’s ρ=0.76).

## Discussion

This study evaluated the validity of WBE as a surveillance tool for monitoring norovirus in BC by comparing wastewater concentrations to hospital admissions and ED visits over a 4.75-year period. While clinical indicators underestimate true disease burden and are an imperfect comparator, wastewater signals correlate with well with both hospital admissions and ED visits. This indicates that norovirus levels in wastewater reflect real-world illness trends and may serve as a valid proxy for disease incidence where individual cases are not reportable. This is consistent with the findings of previous research and adds to the evidence supporting the use of WBE for norovirus surveillance (18–32).

Time lagged cross-correlations showed that increases in wastewater signals preceded increases in norovirus-related hospital admissions by approximately two weeks, suggesting that wastewater may provide an early warning of severe norovirus disease. This may afford healthcare facilities critical lead time to consider resource allocation and implement strategies to prevent further transmission, such as enhanced screening and cleaning protocols. Conversely, the highest correlation between wastewater signals and ED visits occurred when neither was shifted (unadjusted wastewater) or when ED visits preceded wastewater by one week, indicating that both occur around the same time, with wastewater signals potentially lagging ED visits.

This discrepancy may be attributable to several factors. Emergency department visits tend to capture initial healthcare seeking early in the course of illness, whereas admissions represent more severe cases with complications that develop following norovirus infection. Emergency department visits may therefore align more closely with community transmission at any given time. The use of down sampling to reduce the data to a weekly frequency may also have masked lead time within the ED visit data itself, particularly given the short incubation period of norovirus, which ranges from 12 to 72 hours (4). Indeed, norovirus shedding has been found to peak around 2–5 days after infection, suggesting that wastewater detection is likely to occur around the same time as or even after symptom onset, potentially limiting the ability of WBE to provide an early warning for this pathogen. Furthermore, the lack of observed lag between wastewater signals and ED visits versus hospitalizations may reflect the quality of the clinical data itself, which captures GI-related illness more broadly, as opposed to norovirus specifically. Nausea, vomiting and diarrhea can have a range of etiologies, both infectious and non-infectious, and a recent study looking at children presenting to ED with GI symptoms found that only 36.5% tested positive for norovirus on a rectal swab or stool culture (40). The data had a baseline of at least 500 GI-related ED visits per week and this background noise may reduce temporal precision, making it difficult to detect a lead-lag relationship even where one truly exists.

### Limitations

Our findings should be interpreted with several limitations in mind. First, the lack of gold-standard norovirus case data drove us to use hospital indicators, which are incomplete and are sensitive to changes in care-seeking behaviours. For example, the study coincided with the COVID-19 pandemic, which led to a 15% reduction in ED visits in BC (41). While GI-related visits had largely returned to baseline by mid-2021 (41), the clinical data may not provide a stable estimate of GI illness across study years. Second, norovirus-related hospital admissions were identified using ICD-10-CA diagnostic code A08.1, which captures both noroviral enteritis and small round structured virus enteritis (42). As a result, some of the hospitalizations included in this analysis may not be due to norovirus but to other, similar viruses, which may follow a different seasonal pattern. Third, the populations captured by the wastewater and clinical data sources used in this study do not always overlap. The WWTPs sampled were predominantly located in urban areas in southern BC, and the ED visit data were similarly sourced from a subset of 30 primarily urban acute care facilities. In contrast, hospital admissions data were reported by all facilities across the province. Differences in population coverage may have influenced the magnitude or timing of the observed associations. Similarly, while generally thought to provide a good approximation of community-level trends, wastewater surveillance is not able to capture fecal contributions from certain populations, such as those who use diapers. Given that norovirus commonly affects infants and young children, and that vomiting is a more prominent symptom than diarrhea in this age group (4), wastewater data may underestimate the true burden of norovirus. Finally, by aggregating data for the whole province, the analysis may have masked regional differences in the strength and direction of the association between wastewater and clinical indicators at smaller spatial resolutions. Future research should assess how the wastewater data and clinical indicators correlate sub-provincially and over different temporal periods to better understand their value in capturing and predicting local norovirus activity.

## Conclusion

Like many non-reportable diseases, norovirus is highly underrepresented in existing surveillance databases and clinical indicators capture only the most severe cases that require medical intervention, providing a poor approximation of population-level burden of illness. The findings demonstrate the value of WBE, suggesting that it serves as a valid measure of disease trends and can be used to monitor norovirus activity and inform the implementation of norovirus prevention and education strategies. While its usefulness for predicting GI-related ED visits remains unclear, the results suggest WBE may also serve as an early warning indicator for severe disease requiring hospitalization, enabling health system planning and preparedness. As such, the results strengthen confidence in WBE as a strategy for reducing gaps in norovirus surveillance in BC and beyond.

## Appendix

Supplemental material is available upon request to the author: bridget.irwin@bccdc.ca
